# Supplementary material for: Effects of social distancing on the spreading of COVID-19 inferred from mobile phone data
Source: Sci Rep. 2021 Jan 18;11:1661. doi: 10.1038/s41598-021-81308-2 (PMC7813880; doi:10.1038/s41598-021-81308-2)
Supplement: Supplementary file 1 — Supplementary Information. [file 41598_2021_81308_MOESM1_ESM.pdf]

Effects of social distancing on the spreading of COVID-19 inferred from mobile phone data

Hamid Khataee[1], Istvan Scheuring[2,3], Andras Czirok[4,5], and Zoltan Neufeld[1,*]

[1]School of Mathematics and Physics, The University of Queensland, St. Lucia, Brisbane, QLD 4072, Australia
[2]Evolutionary Systems Research Group, Centre for Ecological Research, Tihany, 8237, Hungary
[3]MTA-ELTE Theoretical Biology and Evolutionary Ecology Research Group, Eotvos University, Budapest, Hungary
[4]Department of Biological Physics, Eotvos University, Budapest, 1053, Hungary
[5]Department of Anatomy and Cell Biology, University of Kansas Medical Center, Kansas City, KS 66160, USA
[*]Corresponding author: z.neufeld@uq.edu.au

December 2, 2020

1 Supplemental Material

Table S1: Calendar dates associated with the daily deaths and mobility data.

**Country**	**Time 0**	t_{NL}	t_{eff}
ITA	March 3	March 12	March 3
ESP	March 11	March 14	March 11
FRA	March 12	March 17	March 13
GBR	March 16	March 23	March 15
GER	March 20	March 23	March 12
BEL	March 22	March 17	March 11
NLD	March 18	March 23	March 11
SWL	March 21	March 13	March 11
SWE	March 27	-	March 10

Time 0: the day when a country first reported ≥ 5 daily deaths. t_{NL}: national lock-down date. t_{eff}: effective lock-down date.

Table S2: Parameters from fitting Equation (1), to the number of daily deaths data during the exponential growth and exponential decay phases. Parameter values correspond to mean ± SE.

**Country**	D_{0_1}	α_1	D_{0_2}	α_2
ITA	9.934 ± 0.86	0.25 ± 0.008	2207.08 ± 75.03	-0.032 ± 0.001
ESP	9.818 ± 0.79	0.25 ± 0.006	2987.16 ± 321.018	-0.046 ± 0.003
FRA	6.95 ± 0.50	0.22 ± 0.006	6970.80 ± 388.52	-0.058 ± 0.001
GBR	8.59 ± 0.81	0.22 ± 0.006	2739.04 ± 89.85	-0.030 ± 0.001
GER	7.32 ± 0.47	0.20 ± 0.006	1089.01 ± 48.01	-0.046 ± 0.001
BEL	9.97 ± 0.61	0.20 ± 0.006	1433.01 ± 69.99	-0.056 ± 0.001
NLD	7.28 ± 0.31	0.22 ± 0.004	851.46 ± 49.79	-0.052 ± 0.001
SWL	5.25 ± 0.46	0.19 ± 0.010	321.90 ± 25.24	-0.064 ± 0.002
SWE	10.53 ± 0.61	0.14 ± 0.006	151.46 ± 11.16	-0.018 ± 0.001

Table S3: Parameters from fitting Equation (3) to the mobility data over a period of 90 days starting from 13-January-2020. Parameter values correspond to mean ± SE.

**Country**	M_1	t_1	M_2	t_2
ITA	116.07 ± 1.74	-11.00 ± 1.03	14.45 ± 2.08	11.01 ± 1.06
ESP	123.87 ± 1.94	-2.75 ± 0.72	10.80 ± 2.77	3.51 ± 0.69
FRA	101.22 ± 1.24	-1.69 ± 0.62	16.27 ± 1.87	4.63 ± 0.62
GBR	115.37 ± 1.51	-6.84 ± 1.09	36.64 ± 2.51	5.20 ± 1.05
GER	112.40 ± 1.30	-13.44 ± 1.05	46.14 ± 1.96	-1.21 ± 1.12
BEL	126.14 ± 1.74	-13.81 ± 0.93	39.36 ± 2.52	-6.37 ± 0.91
NLD	112.78 ± 1.22	-11.78 ± 0.87	39.86 ± 1.78	-1.40 ± 0.87
SWL	108.10 ± 1.00	-15.25 ± 0.80	44.72 ± 1.46	-3.28 ± 0.87
SWE	112.53 ± 0.95	-21.00 ± 1.18	70.60 ± 1.33	-12.06 ± 1.19

M_1: average mobility level before social distancing. t_1: when the mobility started to decrease due to social distancing. M_2: average mobility after the social distancing. t_2: end of the transition period of social distancing.
